# Targeting *KRAS*‐mutant pancreatic cancer through simultaneous inhibition of KRAS, MEK, and JAK2

**DOI:** 10.1002/1878-0261.13751

**Published:** 2024-10-14

**Authors:** Satoru Miyazaki, Masato Kitazawa, Satoshi Nakamura, Makoto Koyama, Yuta Yamamoto, Nao Hondo, Masahiro Kataoka, Hirokazu Tanaka, Michiko Takeoka, Daisuke Komatsu, Yuji Soejima

**Affiliations:** ^1^ Division of Gastroenterological, Hepato‐Biliary‐Pancreatic, Transplantation and Pediatric Surgery, Department of Surgery Shinshu University School of Medicine Matsumoto Japan; ^2^ Department of Surgery Jinai Hospital Ina Japan

**Keywords:** fedratinib, KRAS, MRTX1133, pancreatic cancer, sotorasib, trametinib

## Abstract

The Kirsten rat sarcoma (*KRAS*) oncogene was considered “undruggable” until the development of sotorasib, a KRAS^G12C^ selective inhibitor that shows favorable effects against lung cancers. MRTX1133, a novel KRAS^G12D^ inhibitor, has shown promising results in basic research, although its effects against pancreatic cancer are limited when used alone. Therefore, there is an urgent need to identify effective drugs that can be used in combination with KRAS inhibitors. In this study, we found that administration of the KRAS inhibitors sotorasib or MRTX1133 upregulated STAT3 phosphorylation and reactivated ERK through a feedback reaction. The addition of the MEK inhibitor trametinib and the JAK2 inhibitor fedratinib successfully reversed this effect and resulted in significant growth inhibition *in vitro* and *in vivo*. Analyses of sotorasib‐ and MRTX1133‐resistant cells showed that trametinib plus fedratinib reversed the resistance to sotorasib or MRTX1133. These findings suggest that the JAK2‐mediated pathway and reactivation of the MAPK pathway may play key roles in resistance to KRAS inhibitors in pancreatic cancers. Accordingly, simultaneous inhibition of KRAS, MEK, and JAK2 could be an innovative therapeutic strategy against *KRAS*‐mutant pancreatic cancer.

## Graphical abstract

The authors found that administration of the KRAS inhibitors sotorasib or MRTX1133 to pancreatic cancer cell lines upregulated STAT3 phosphorylation and reactivated ERK through a feedback reaction. Addition of the MEK inhibitor trametinib and the JAK2 inhibitor fedratinib successfully reversed this effect and resulted in significant growth inhibition *in vitro* and *in vivo*.

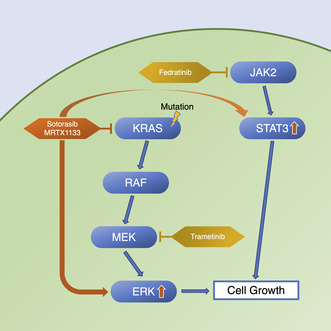

**Abbreviations**
AKT: protein kinase B
ANOVA: analysis of variance
CCK‐8: cell counting kit‐8
EGFR: epidermal growth factor receptor
ERK: extracellular signal‐regulated kinase
GEM‐NABP: gemcitabine with nab‐paclitaxel
IHC: immunohistochemical
JAK2: Janus kinase 2
KRAS: Kristen rat sarcoma
MEK: mitogen‐activated protein kinase
NRTK: neurotrophic tropomyosin‐receptor kinase
PDAC: pancreatic ductal adenocarcinoma
PI3K: phosphatidylinositol‐3 kinase
RTK: receptor tyrosine kinase
STAT3: signal transducer and activator of transcription 3
TRK: tropomyosin‐related kinase

## 1 Introduction

Pancreatic cancer is the third leading cause of cancer‐related deaths in the United States and fourth in Japan [1, 2]. According to the latest Japanese statistics, the 5‐year survival rate for pancreatic cancer is only 12.1%, which is significantly lower than that for other cancers such as bile duct cancer (28.7%), esophageal cancer (50.1%), and colorectal cancer (76.8%) [2]. While only 20% of patients with pancreatic cancer undergo curative resection [3], effective molecule‐based therapies against pancreatic cancer are lacking. Treatment with FOLFIRINOX, a combination of fluorouracil, leucovorin, irinotecan, oxaliplatin, and gemcitabine with nab‐paclitaxel (GEM‐NABP), has shown superior effects in patients with pancreatic cancer when compared to those treated with gemcitabine alone [4]. However, median progression‐free survival remains limited, standing at 7.3 months with FOLFIRINOX and 5.7 months with GEM‐NABP [5, 6]. The epidermal growth factor receptor (EGFR) inhibitor erlotinib has an additive effect on gemcitabine and is clinically available for use, but the effect is not remarkable, merely elevating progression‐free survival from 3.55 to 3.75 months [7].

Pancreatic cancer cells harbor several gene mutations during the course of cancer development, with Kirsten rat sarcoma (*KRAS*) mutations accounting for 94.1%, followed by *TP53* (63.9%), *SMAD4* (20.8%), and *CDKN2A* mutations (17.0%) [8]. Patients with pancreatic cancer harboring *BRCA1*/*2* mutations can benefit from olaparib [9], a clinically available poly ADP ribose polymerase (PARP) inhibitor. However, mutations in *BRCA1*/*2* are reported in only 4–7% of patients [10], making olaparib a relatively rare treatment option. Larotrectinib, an inhibitor of tropomyosin‐related kinase (TRK), is clinically available for neurotrophic tropomyosin‐receptor kinase (*NRTK*) fusion‐positive patients with pancreatic ductal adenocarcinoma (PDAC) [11]; however, the positive rate of *NRTK* fusion is less than 1% in patients [12], thereby limiting the pool of patients with available treatment options. Therefore, the development of molecular‐targeting therapies for more frequent genetic mutations is crucial to maximize the number of patients who can benefit. Furthermore, there is an urgent need to develop effective molecular therapies that can facilitate conversion surgery and long‐term survival.

*KRAS* is one of the most frequently mutated oncogenes associated with human cancers. Approximately 90% of pancreatic cancers exhibit *KRAS* mutations, and approximately 40% of these cases harbor the *KRAS* ^*G12D*^ mutation [8]. Although targeting KRAS in pancreatic cancer is an attractive strategy because of the high frequency of mutations compared to that in other cancers, the development of KRAS inhibitors has remained unsuccessful for decades. Progress in the development of KRAS inhibitors is particularly crucial because successful implementation of KRAS‐targeted therapy could revolutionize treatment outcomes, especially considering the high malignancy of pancreatic cancers. A recently developed covalent KRAS^G12C^‐selective inhibitor, sotorasib, is now available for clinical use in *KRAS* ^*G12C*^‐mutant lung cancer treatment [13]. Additionally, a novel noncovalent KRAS^G12D^ selective inhibitor, MRTX1133, has demonstrated promising effects in basic research [14, 15]. However, resistance to KRAS inhibitors when used alone has been reported in several cancers other than lung cancer [16], highlighting the urgency to discover effective drugs for combination therapy.

Several acquired resistance mechanisms of KRAS^G12C^ inhibitors have been reported, including acquired *KRAS* alterations besides G12C and amplification of the *KRAS* ^*G12C*^ allele; *MET* amplification; mutations in *NRAS*, *BRAF*, *MAP2K1*, and *RET*; fusions in *ALK*, *RET*, *BRAF*, *RAF1*, and *FGFR3*; and loss‐of‐function mutations in *NF1* and *PTEN* [17]. In colon cancers, rapid adaptive feedback reactivation driven by receptor tyrosine kinases (RTK)‐mediated activation of wild‐type KRAS has been reported following KRAS^G12C^ inhibition and co‐inhibition of SHP2 overcomes this resistance [18]. Adagrasib, another KRAS^G12C^ inhibitor, is currently under clinical trials. Adagrasib combined with the EGFR receptor inhibitor cetuximab showed a higher response rate than monotherapy [19], which emphasizes the need for effective agents in combination with KRAS inhibitors. A previous study has shown that feedback reactivation of the RAF–MEK–ERK (MAPK) pathway is vital for sotorasib resistance in *KRAS* ^*G12C*^‐mediated colon cancer [20]. Furthermore, signal transducer and activator of transcription 3 (STAT3) is essential for resistance to mitogen‐activated protein kinase (MEK) inhibition in PDAC cell lines [21]. This study aimed to identify the molecules that regulate resistance to KRAS inhibitors in PDAC and explore whether simultaneous targeting of these molecules could be an effective therapy to overcome that resistance.

## 2 Materials and methods

### 2.1 Cell culture

MIA PaCa‐2 (*KRAS* ^*G12C*^, RRID: CVCL_0428), SUIT‐2 (*KRAS* ^*G12D*^, RRID: CVCL_3172), and KP‐4 (*KRAS* ^*G12D*^, RRID: CVCL_1338) cells were purchased from JCRB (Osaka, Japan). PANC‐1 (*KRAS* ^*G12D*^, RRID: CVCL_0480) and PK‐59 (*KRAS* ^*G12D*^, RRID: CVCL_4897) cells were purchased from RIKEN (Ibaraki, Japan).

MIA PaCa‐2 cells were maintained in Dulbecco's modified Eagle's medium (DMEM; low‐glucose; Fujifilm WAKO, Osaka, Japan). SUIT‐2, KP‐4, PANC‐1, and PK‐59 cells were maintained in RPMI‐1640 medium (Fujifilm WAKO). All media were supplemented with 10% fetal bovine serum (Biowest, Nuaillé, France) and 1% penicillin–streptomycin (Fujifilm WAKO).

Cell lines used in this study were authenticated by short tandem repeat profiling. All the experiments were performed with mycoplasma‐free cells.

### 2.2 Antibodies and reagents

The following antibodies were used: monoclonal rabbit p‐ERK1/2 (#4376, RRID: AB_331772; Cell Signaling Technology [CST], Danvers, MA, USA), monoclonal rabbit ERK1/2 (#4695, RRID: AB_390779; CST), monoclonal rabbit p‐AKT (#4060, RRID: AB_2315049; CST), monoclonal rabbit AKT (#4691, RRID: AB_915783; CST), monoclonal rabbit p‐STAT3 (#9145, RRID: AB_2491009; CST), monoclonal rabbit STAT3 (#4904, RRID: AB_331269; CST), β‐actin (#sc‐47 778, RRID: AB_2714189; Santa Cruz Biotechnology, Dallas, TX, USA), and Ki‐76 (#SAB5700770; Sigma‐Aldrich, St. Louis, MO, USA). The secondary antibodies were polyclonal goat anti‐mouse IgG (#P0447, RRID: AB_2617137; Dako, Agilent Technologies, Santa Clara, CA, USA) and polyclonal goat anti‐rabbit IgG (#P0448, RRID: AB_2617138; Dako, Agilent Technologies) conjugated with HRP.

Sotorasib, MRTX1133, trametinib, and fedratinib were purchased from Selleck Chemicals (Houston, TX, USA).

### 2.3 Cell proliferation assay and calculation of the combination index

Cell proliferation was evaluated using the Cell Counting Kit‐8 (CCK‐8) (#343‐07623; Dojindo Laboratories, Kumamoto, Japan). Cells were seeded into 96‐well tissue culture plates and incubated at 37 °C to allow adhesion. After 24 h, each reagent or Dimethyl sulfoxide (DMSO) (control) was added to individual wells. The CCK‐8 reagent was added to the wells 72 h after the addition of reagents according to the manufacturer's instructions and incubated at 37 °C for 2 to 4 h. The absorbance was measured at 450 nm using a BioTek Epoch microplate reader (Agilent Technologies), and cell viability was calculated as the ratio of the absorbance of test wells to that of control wells. Synergy analyses were performed using the combination index calculated via the Chou‐Talalay method, which is based on the median effect equation [22]. The combination index was calculated using the compusyn software (ComboSyn Inc., Paramus, NJ, USA, version 1.0.1). A combination index below 0.7 was considered to indicate synergistic effects [23].

### 2.4 Protein sample preparation and western blotting

After drug treatment and incubation, the cells were washed thrice with cold phosphate‐buffered saline and lysed in the RIPA Lysis Buffer System (#sc‐24948; Santa Cruz Biotechnology) on ice. The lysates were centrifuged at 10 000 ***g*** and 4 °C for 10 min, and the supernatant was collected as a whole‐cell lysate. Proteins were quantified using the Pierce BCA Protein Assay Kit (#23227; Thermo Fisher Scientific, Waltham, MA, USA). NuPAGE 4–12% gels (# NP0322; Thermo Fisher Scientific) were used to separate 10–25 μg protein, and the resolved proteins were electroblotted onto Polyvinylidene fluoride membranes. After blocking with Tris‐buffered saline containing 5% nonfat dry milk and 0.1% Tween‐20 for 1 h at 20–24 °C, the membranes were probed with primary antibodies overnight at 4 °C and secondary antibodies conjugated to horseradish peroxidase for 1 h at 20–24 °C. The bands were detected via chemiluminescence using Amersham ECL Select Western Blotting Detection Reagent (GE Healthcare Life Sciences, Chicago, IL, USA) and photographed using the ChemiDoc XRS device (Bio‐Rad Laboratories, Hercules, CA, USA). The integrated density of the target protein was quantified using image lab Software version 6.1 for Mac (Bio‐Rad Laboratories).

### 2.5 Establishment of sotorasib‐ and MRTX1133‐resistant cells

Sotorasib‐resistant MIA PaCa‐2 cells were generated via continuous exposure to sotorasib. The initial concentration of sotorasib was 10 nm, and the concentration was gradually increased to 100 nm over 2 months. Resistant cell lines were maintained at 100 nm of sotorasib and passaged 10 times before analysis. MRTX1133‐resistant cell lines were established from SUIT‐2 and KP‐4 cells by the same method.

### 2.6 *In vivo* xenograft experiments

All animal experiments were approved by the Institutional Animal Care and Use Committee of Shinshu University (approval no. 023005) and performed in compliance with the Guide for the Care and Use of Laboratory Animals (eighth edition, National Research Council). Six‐week‐old male BALB/cAJcl*Foxn1* ^*nu*^ mice were purchased from CLEA Japan (Shizuoka, Japan) and maintained in a specific pathogen‐free room with a 12‐h light/dark cycle and free access to water and food. Xenograft tumors were generated via subcutaneous injection of SUIT‐2 cells and MRTX1133‐resistant SUIT‐2 cells (1.0 × 10^7^), suspended in 200 μL of 50% Hank's Balanced Salt Solution (FUJIFILM Wako), into the flanks of mice. Vehicle (5% DMSO + 40% PEG 300 + 5% Tween 80 + 50% ddH_2_O), MRTX1133 (0.5 mg·kg^−1^ for SUIT‐2 cells and 2.0 mg·kg^−1^ for MRTX1133‐resistant SUIT‐2 cells), trametinib (0.1 mg·kg^−1^), fedratinib (25 mg·kg^−1^), or a combination of the three above‐mentioned drugs were orally administered daily for 21 days. Tumors were measured twice a week to determine tumor volume using the following formula:

Volume=length×width×height×π/6.

Mice were euthanized on day 22 via cervical dislocation under anesthesia, and the tumors were surgically resected, weighed, and fixed in a 10% formalin neutral buffer solution (Fujifilm WAKO) for immunohistochemical (IHC) examination.

### 2.7 IHC

The resected xenograft tumors were fixed in a 10% formalin neutral buffer solution (Fujifilm WAKO) for 16 h at 20–24 °C and embedded in paraffin. Then, 4‐μm‐thick sections were prepared for immunostaining. The sections were boiled at 98 °C for 25 min in a 1 mm EDTA2Na solution (pH 9.0) for antigen retrieval. Endogenous peroxidase activity was blocked using 3% of H_2_O_2_. Slides were incubated with primary antibodies for 16 h at 4 °C in a humidified chamber. Histofine Simplestain MAX PO (Nichirei Bioscience, Tokyo, Japan) was used as the secondary antibody according to the manufacturer's instructions. DAB was used for visualization, and hematoxylin was used for counterstaining. For TUNEL staining, an *in situ* apoptosis detection kit (Takara Bio Inc. Kusatsu, Shiga, Japan) was used according to the manufacturer's instructions; a methyl green solution (Fujifilm WAKO) was used for counterstaining. All the slides were visualized and imaged by an CKX53 optical microscope (Olympus, Tokyo, Japan).

### 2.8 Statistical analysis

Data were obtained from three or more independent experiments. All statistical analyses were performed using EZR (Saitama Medical Center, Jichi Medical University, Saitama, Japan, version 1.61), which is a graphical user interface for r (The R Foundation for Statistical Computing, Vienna, Austria, version 4.2.2). Statistical significance was evaluated using an unpaired Student's *t*‐test or a one‐way analysis of variance (ANOVA), followed by Tukey's test. Data are presented as mean ± standard deviation. Statistical significance was set at *P* < 0.05.

## 3 Results

### 3.1 Feedback activation of the MEK/ERK, JAK2/STAT3, and PI3K/AKT pathways occurs following treatment with sotorasib in *KRAS* ^*G12C*^ PDAC cell lines

**Fig. 1:**
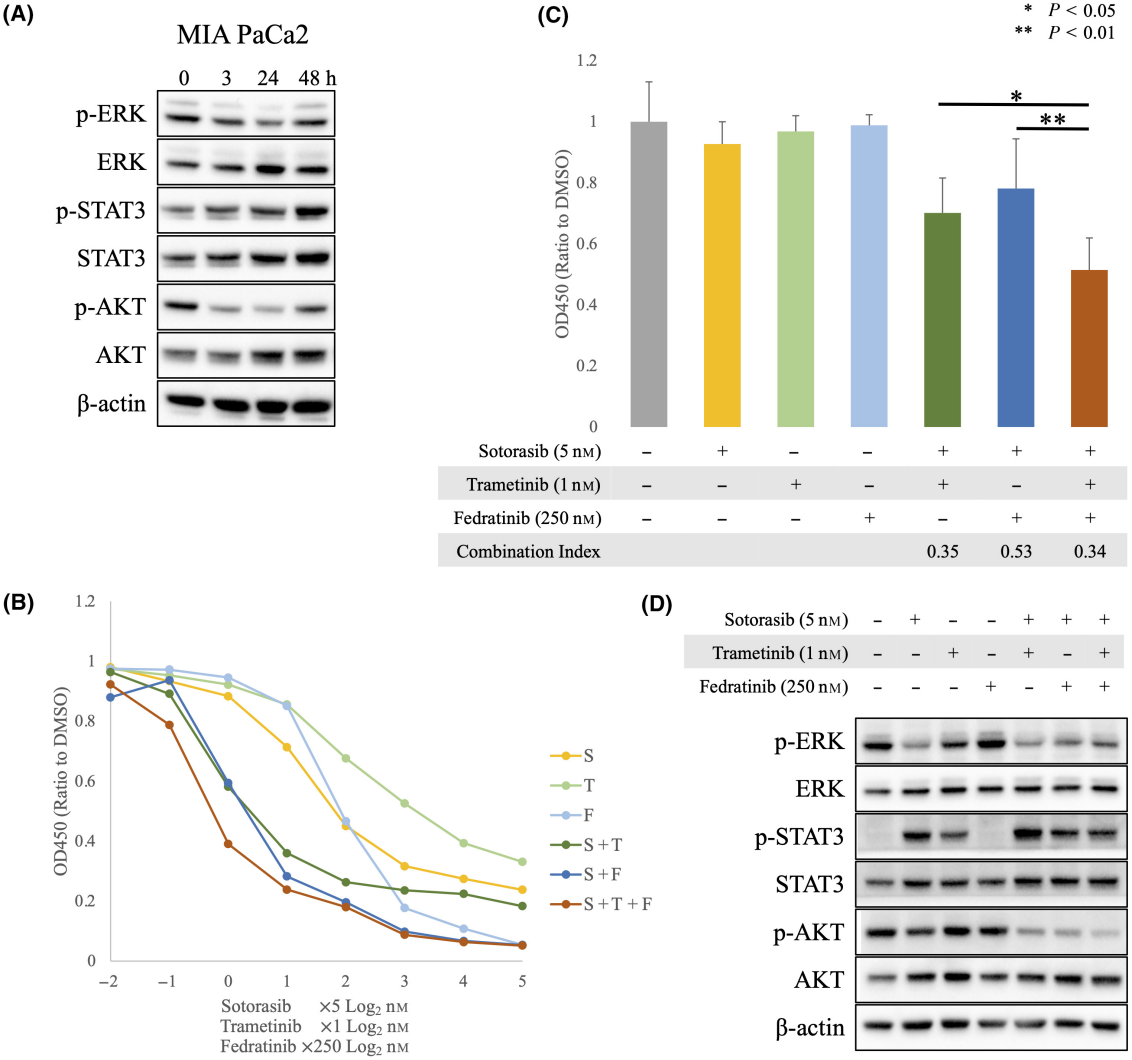
Feedback activation of ERK and STAT3 occurs following treatment with sotorasib in *KRAS* ^*G12C*^ pancreatic ductal adenocarcinoma (PDAC) cell lines. (A) *KRAS* ^*G12C*^ PDAC cell line MIA PaCa‐2 was treated with 5 nm sotorasib for 0, 3, 24, and 48 h. The expression of proteins involved in the RAS/MAPK, JAK2/STAT3, and PI3K/AKT pathways was evaluated using western blotting analysis. A representative image is shown (*n* = 4). (B) Dose–response curve of sotorasib, trametinib, fedratinib, and their combinations established using Cell Counting Kit‐8 (CCK‐8). S: sotorasib; T: trametinib; F: fedratinib (*n* = 8). (C, D) MIA PaCa‐2 was treated with sotorasib (5 nm), trametinib (1 nm), and/or fedratinib (250 nm). (C) Proliferation was assessed using CCK‐8 (*n* = 8). Statistical significance was evaluated using a one‐way ANOVA followed by Tukey's test. **P < 0.01, *P < 0.05. Error bars indicate standard deviations. (D) Cell lysates for western blotting analysis were collected 48 h after treatment. A representative image is shown (*n* = 3).

To understand the mechanisms of resistance to KRAS inhibitors in PDAC, we first assessed the effects of sotorasib on the MEK/ERK, JAK2/STAT3, and PI3K/AKT pathways. The *KRAS* ^*G12C*^ PDAC cell line MIA PaCa‐2 was treated with sotorasib, and cell lysates were collected at different time points (3–48 h). Western blotting analysis showed that ERK and AKT expressions were suppressed after 3 h of treatment; however, consistent with our previous reports on colon cancer [20], ERK and AKT were reactivated 48 h post‐treatment. Interestingly, phosphorylation of STAT3 did not change in the short term but gradually increased after 48 h of treatment (Fig. 1A).

### 3.2 MEK inhibitor and JAK2 inhibitor enhance the efficacy of sotorasib *in vitro*

Next, we evaluated whether the co‐inhibition of MEK by trametinib and JAK2 by fedratinib suppresses ERK and STAT3 activation through a feedback mechanism to overcome sotorasib resistance. The results of the proliferation assay revealed the synergistic effects of the combination of the two drugs at concentrations that were less effective as single agents (Fig. 1B). The three‐drug combination of sotorasib, trametinib, and fedratinib showed an even stronger inhibitory effect than the combination of the two drugs; the combination index was the lowest, at 0.34, for the three‐drug combination therapy, confirming the synergistic effect of the three drugs (Fig. 1C and Fig. S1B). Western blotting analysis showed that inhibition of the RAS/MAPK pathway activated STAT3, and inhibition of the JAK2/STAT3 pathway activated ERK. Thus, the three‐drug combination therapy successfully suppressed the phosphorylation of ERK and STAT3 compared to sotorasib alone (Fig. 1D).

### 3.3 Reactivation of the MEK/ERK and JAK2/STAT3 pathways, but not the PI3K/AKT pathway, occurs following treatment with MRTX1133 in the *KRAS* ^*G12D*^ PDAC cell lines

**Fig. 2:**
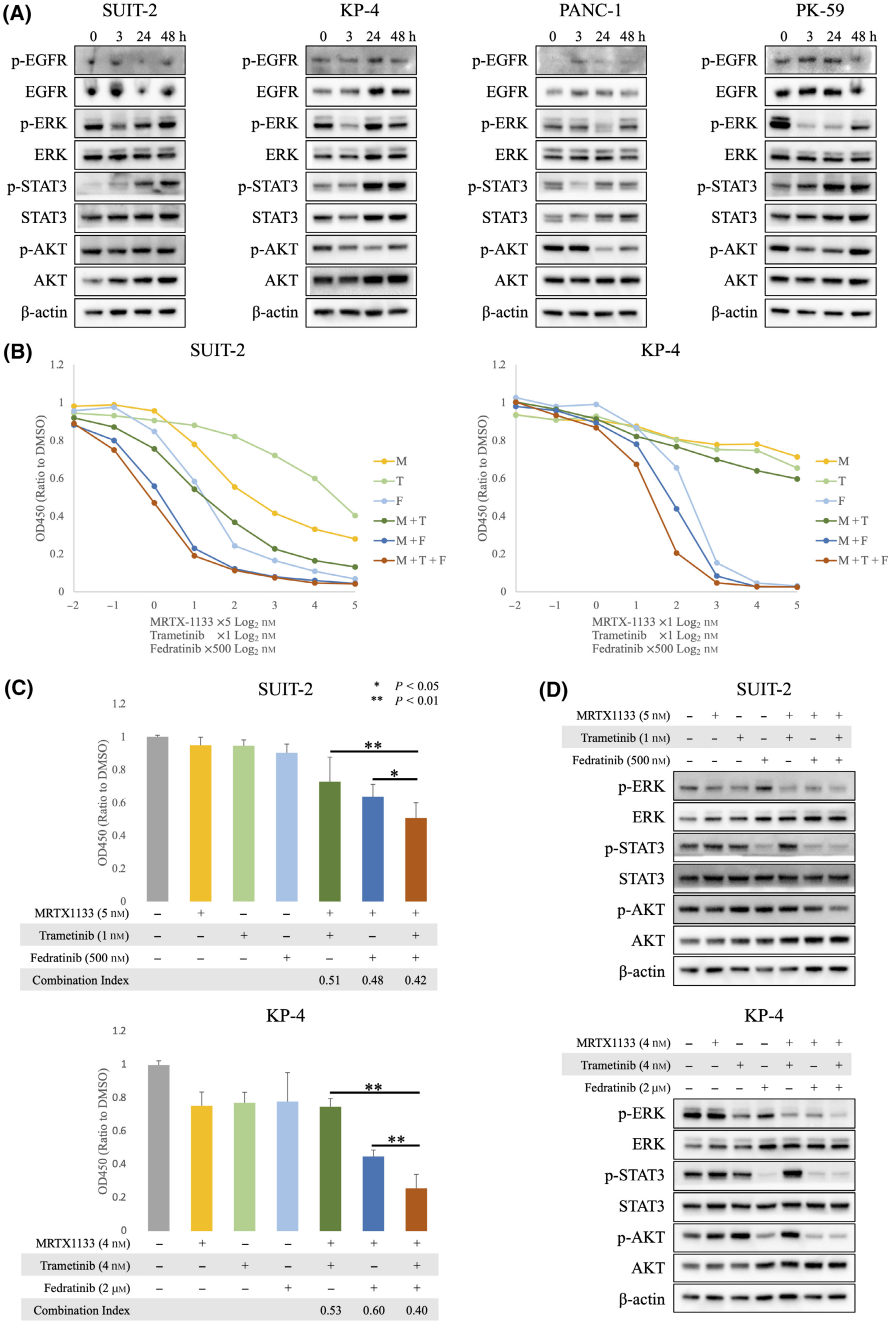
Combination of MRTX1133, an MEK inhibitor, and a JAK2 inhibitor is effective in *KRAS* ^*G12D*^ mutant PDAC cell lines. (A) The *KRAS* ^*G12D*^ cell lines SUIT‐2, KP‐4, PANC‐1, and PK‐59 were treated with 5 nm MRTX1133 for 0, 3, 24, and 48 h. The expression of proteins involved in the RAS/MAPK, JAK2/STAT3, and PI3K/AKT pathways was evaluated using western blotting analysis. Representative image is shown (*n* = 3). (B) Dose–response curve of MRTX1133, trametinib, fedratinib, and their combinations established using Cell Counting Kit‐8 (CCK‐8). M: MRTX1133; T: trametinib; F: fedratinib. (C, D) SUIT‐2 was treated with MRTX1133 (5 nm), trametinib (1 nm), and/or fedratinib (500 nm) (*n* = 11). KP‐4 was treated with MRTX1133 (4 nm), trametinib (4 nm), and/or fedratinib (2 μm) (*n* = 5). (C) Proliferation of SUIT‐2 (*n* = 11) and KP‐4 (*n* = 5) was assessed using CCK‐8. Statistical significance was evaluated using a one‐way ANOVA followed by Tukey's test. **P < 0.01, *P < 0.05. Error bars indicate standard deviations. (D) Cell lysates were collected 48 h after treatment for western blotting analysis. A representative image is shown (*n* = 3).

We then sought to determine the resistance mechanism of the novel KRAS^G12D^ inhibitor MRTX1133. Consistent with the results obtained with sotorasib, administration of MRTX1133 resulted in similar feedback activation of ERK and STAT3 in *KRAS* ^*G12D*^ PDAC cell lines (Fig. 2A). In contrast, reactivation of AKT, which was observed in the G12C pancreatic cancer cell line MIA PaCA‐2, was only observed in PK‐59 cells treated with MRTX1133. No change was seen in EGFR phosphorylation post‐MRTX113‐treatment in the four *KRAS* ^*G12D*^ PDAC cell lines.

### 3.4 MEK inhibitor and JAK2 inhibitor show synergistic effects in combination with MRTX1133 in *KRAS* ^*G12D*^ PDAC cell lines

We hypothesized that combining MRTX1133 with trametinib, a MEK‐targeting drug, and fedratinib, a JAK2‐targeting agent, would be effective against *KRAS* ^*G12D*^ PDAC. Two cell lines were selected to evaluate the efficacy of the combination therapy. The three‐drug combination therapy showed a significant antitumor effect, as validated by a combination index lower than 0.5 (Fig. 2B,C, and Fig. S1B). Interestingly, KP‐4 showed slight intrinsic resistance to MRTX1133 (Fig. 2B and Fig. S1A); nonetheless, addition of trametinib and fedratinib boosted the inhibitory effect. Western blotting analysis revealed a strong suppression of phosphorylated ERK, STAT3, and AKT levels by the three‐drug combination therapy (Fig. 2D).

### 3.5 MEK inhibitor and JAK2 inhibitor overcome sotorasib and MRTX1133 resistance in resistant cells

**Fig. 3:**
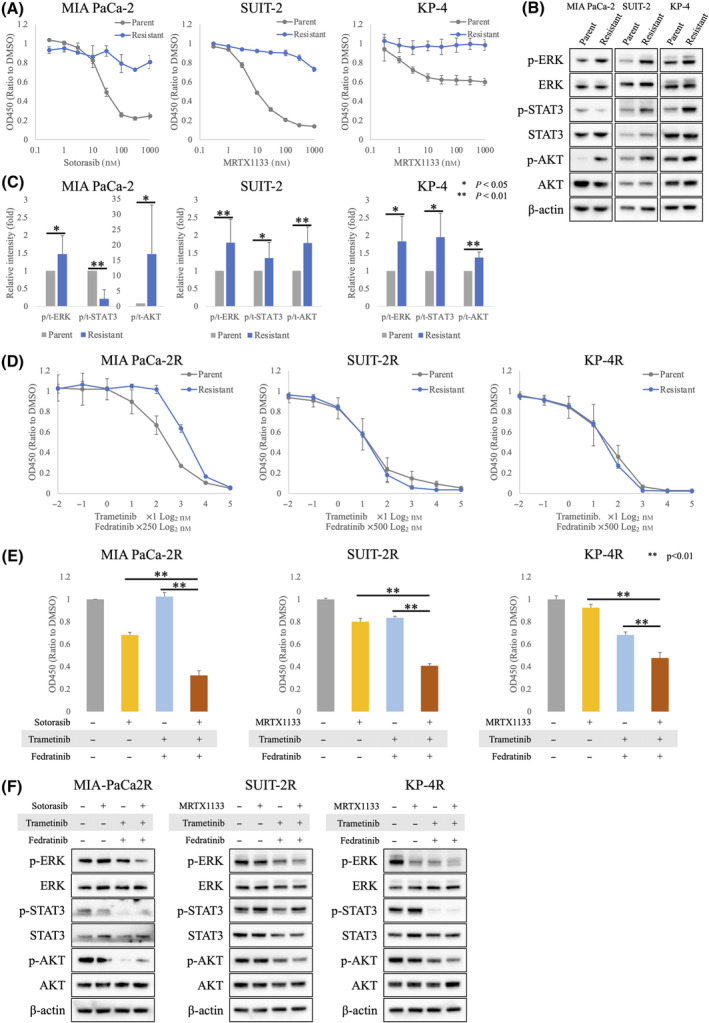
Phosphorylation of ERK and AKT is upregulated in sotorasib‐ and MRTX1133‐resistant cells. (A) Dose–response curve of sotorasib‐ and MRTX1133‐resistant cells and their parental cells established using Cell Counting Kit‐8 (CCK‐8) (*n* = 3). Error bars indicate standard deviations. (B, C) Whole cell lysates were collected for western blotting analysis of the expression of proteins involved in the RAS/MAPK, JAK2/STAT3, and PI3K/AKT pathways. Cells were incubated in sotorasib‐ or MRTX1133‐free media for seven days before the collection of cell lysates. Relative intensity of phosphorylated proteins to that of total proteins of MIA PaCa‐2 (*n* = 5), SUIT‐2 (*n* = 6), and KP‐4 (*n* = 4) were analyzed. Statistical significance was evaluated using the Student's T‐test. **P < 0.01, *P < 0.05. Error bars indicate standard deviations. (D) Dose–response curve of the combination of trametinib and fedratinib in sotorasib‐ or MRTX1133‐resistant cells and their parental cells established using CCK‐8 (*n* = 4). Error bars indicate standard deviations. (E) Proliferation was assessed using CCK‐8 (*n* = 6). Statistical significance was evaluated using the Student's T‐test. **P < 0.01. Error bars indicate standard deviations. (F) Western blotting analysis of cell lysates collected 48 h after drug administration. A representative image is shown (*n* = 3).

To evaluate the mechanisms underlying long‐term resistance to sotorasib and MRTX1133 in PDAC cell lines, we established sotorasib‐resistant cells derived from MIA PaCa‐2 and MRTX1133‐resistant cells derived from SUIT‐2 and KP‐4. All three sotorasib‐resistant or MRTX1133‐resistant cells showed strong resistance to the corresponding KRAS inhibitor, with no inhibitory effect in the nanomolar range (Fig. 3A). Western blotting analysis showed that the phosphorylation of ERK and AKT was significantly increased in all three resistant cells. As expected, the expression of phosphorylated STAT3 was increased in MRTX1133‐resistant SUIT‐2 and KP‐4 cells, whereas it was decreased in sotorasib‐resistant MIA PaCa‐2 cells (Fig. 3B,C). We thought that this might lead to changes in sensitivity to MEK and JAK2 inhibitors. Therefore, we compared the sensitivity of the parental cells and the resistant cells to the combination of trametinib and fedratinib. While the sensitivity to these two drugs did not change in MRTX1133‐resistant SUIT‐2 and KP‐4 cells, sotorasib‐resistant MIA PaCa‐2 cells acquired resistance to the combination of the two drugs (Fig. 3D). Nonetheless, in all three sotorasib‐resistant or MRTX1133‐resistant cells, the addition of trametinib and fedratinib amplified the antitumor effect (Fig. 3E). Similar to that in the parental cells, the three‐drug combination therapy suppressed the phosphorylation of ERK and AKT in resistant cells (Fig. 3F). This suggests that trametinib and fedratinib may overcome resistance to sotorasib and MRTX1133.

### 3.6 Three‐drug combination therapy shows strong antitumor effect *in vivo*

**Fig. 4:**
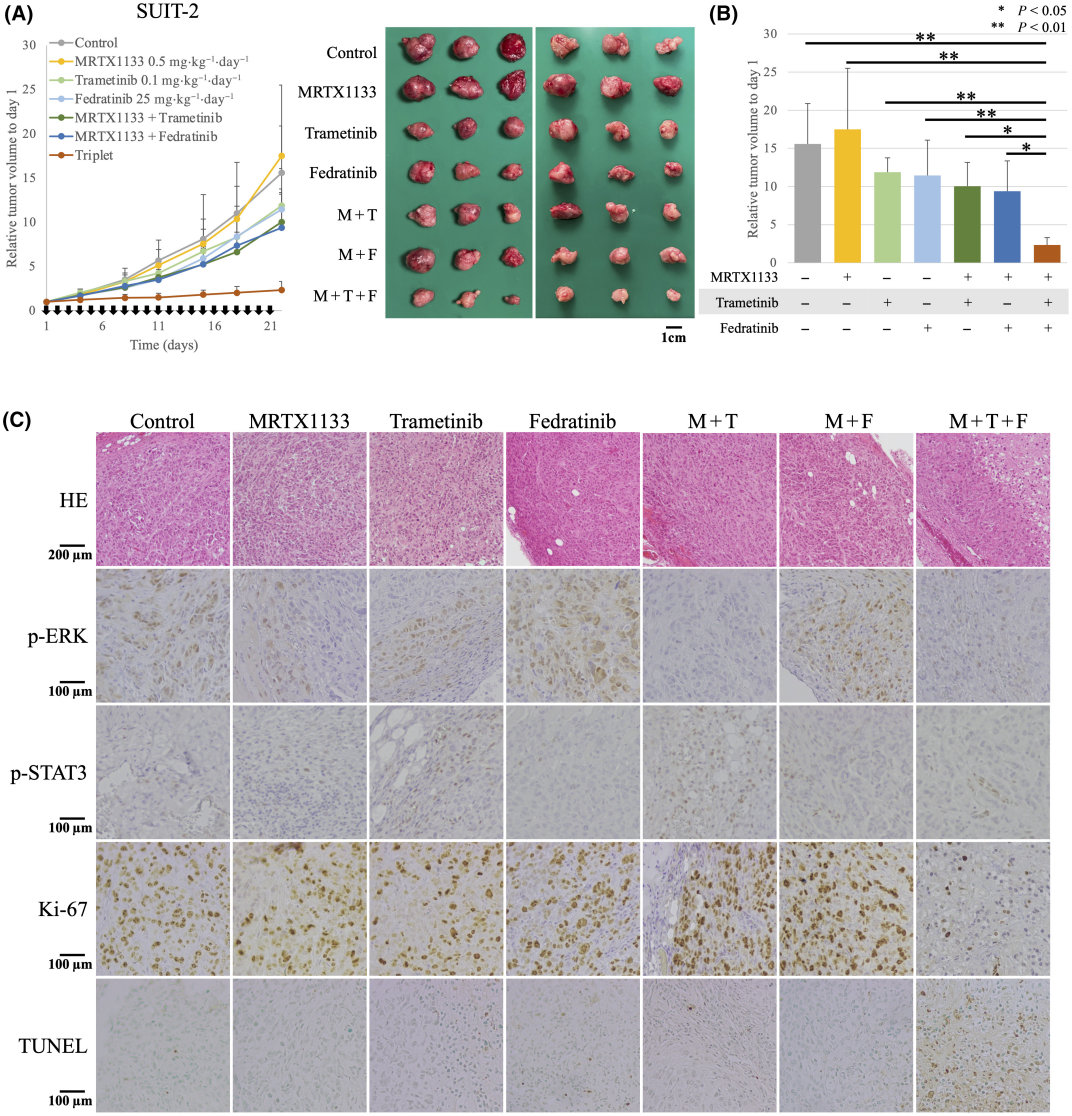
The three‐drug combination has a significantly high tumor inhibition effect *in vivo*. (A) MRTX1133 (0.5 mg·kg^−1^), trametinib (0.1 mg·kg^−1^), and/or fedratinib (25 mg·kg^−1^) were orally administered to BALB/cAJcl*Foxn1* ^*nu*^ mice daily (*n* = 6). Tumor width (W), length (L), and height (H) were measured twice a week. The formula W × L × H × π/6 was used to estimate tumor volume. Relative volume compared with that on day 1 is shown. Error bars indicate standard deviations. Mice were euthanized on day 22. A resected tumor is shown. M: MRTX1133; F: fedratinib; T: trametinib. (B) Relative tumor volume at day 22. Statistical evaluation was performed using one‐way ANOVA followed by Tukey's test. **P < 0.01, *P < 0.05. Error bars indicate standard deviations. (C) The resected tumor was stained for phosphorylated ERK, phosphorylated STAT3, Ki‐67, and TUNEL; a representative image is shown (*n* = 3). M: MRTX1133; F: fedratinib; T: trametinib.

To further evaluate the efficacy of three‐drug combination therapy, murine xenografts of SUIT‐2 cells were generated. Consistent with the results of *in vitro* experiments, each drug used as a single agent showed only limited antitumor effects, whereas the combination of two drugs exhibited a slight antitumor effect. The three‐drug combination therapy resulted in strong tumor suppression throughout the experiment, and significant tumor volume suppression was observed by day 21 (Fig. 4A,B). The resected tumor weight also tended to be the lowest in the three‐drug combination therapy group (Fig. S2B). Immunohistostaining of the resected tumors revealed that the expression of phosphorylated ERK and phosphorylated STAT3 was suppressed, Ki‐67‐positive cells were decreased, and TUNEL‐positive cells were increased in the three‐drug combination therapy group (Fig. 4C). Initial tumor volume at day 1 was 301.3 ± 111.5 mm^3^ (mean ± standard deviation), and no statistically significant difference was seen between the groups (Fig. S2A). Body weight loss was within 10% in all mice throughout the experiment, including the three‐drug combination therapy group (Fig. S2C).

### 3.7 Three‐drug combination therapy shows strong antitumor effect in MRTX1133‐resistant cells *in vivo*

**Fig. 5:**
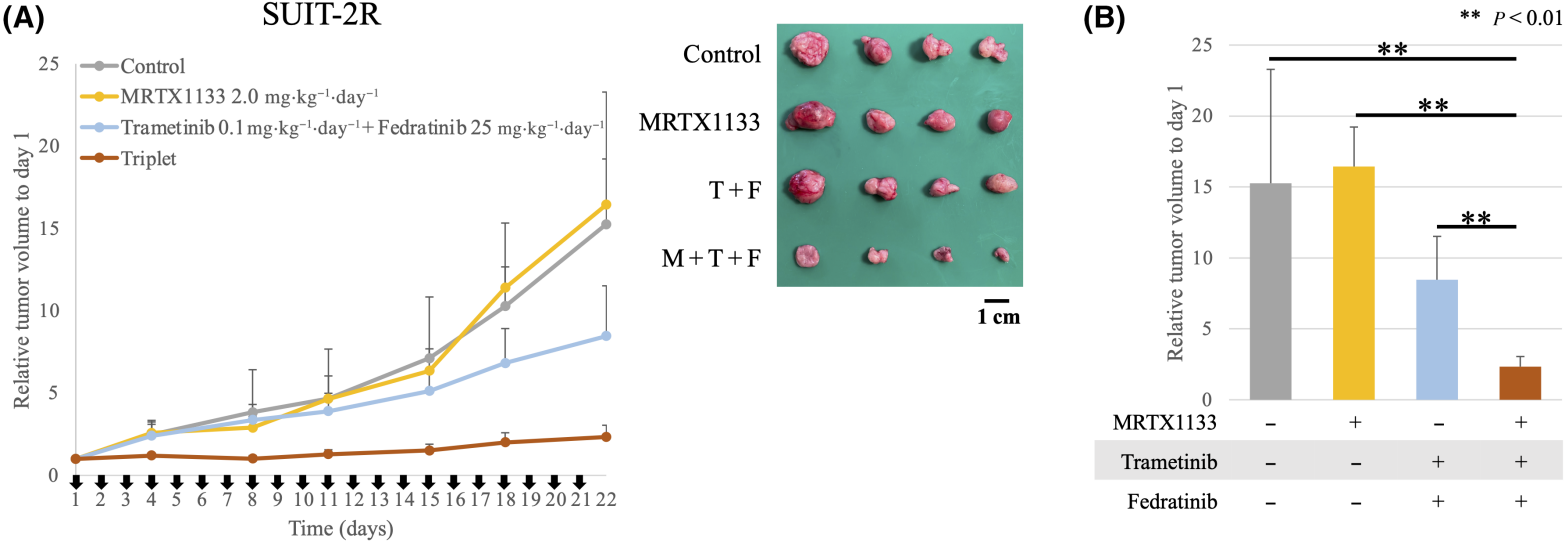
The three‐drug combination also has a strong tumor inhibition effect in MRTX1133‐resistant SUIT‐2 cells *in vivo*. (A) MRTX1133 (2.0 mg·kg^−1^) and/or trametinib (0.1 mg·kg^−1^) plus fedratinib (25 mg·kg^−1^) was orally administered to BALB/cAJcl*Foxn1* ^*nu*^ mice daily (*n* = 4). Tumor width (W), length (L), and height (H) were measured twice a week. The formula W × L × H × π/6 was used to estimate tumor volume. Relative volume compared with that on day 1 is shown. Error bars indicate standard deviations. Mice were euthanized on day 22. A resected tumor is shown. M: MRTX1133; F: fedratinib; T: trametinib. (B) Relative tumor volume at day 22. Statistical significance was evaluated using the Student's T‐test. **P < 0.01. Error bars indicate standard deviations.

To evaluate the efficacy of the three‐drug combination therapy in overcoming long‐term resistance to MRTX1133, murine xenografts of MRTX1133‐resistant SUIT‐2 cells were generated. As with the results of the parental cells, the addition of trametinib and fedratinib overcame the resistance to MRTX1133, showing significant tumor volume suppression throughout the experiment, with the most pronounced tumor volume reduction observed on day 21 (Fig. 5A,B). The resected tumor weight was also the lowest in the three‐drug combination therapy group (Fig. S3B). Initial tumor volume at day 1 was 191.5 ± 118.6 mm^3^ (mean ± standard deviation), and no statistically significant difference was seen between the groups (Fig. S3A). Body weight loss was within 10% in all mice throughout the experiment (Fig. S3C).

## 4 Discussion

Pancreatic cancer is one of the deadliest cancers, with a 5‐year survival rate of 12% [24]. Only 12.9% of pancreatic cancer cases are diagnosed locally [25]. Although surgical resection is the only curative therapy, only 20% of the patients undergo curative resection [3]. A previous study showed that among the few patients who underwent surgery, only 21% survived for 5 years, and 71% experienced recurrence after curative resection [26]. Therefore, the development of an effective chemotherapy is crucial; however, few treatment options are available to date.

Being the most frequently mutated gene in pancreatic cancers, *KRAS* is an attractive target. *KRAS* is mutated in the early stages of pancreatic cancer development; constitutively active *KRAS* is sufficient for the development of PDAC [27]. The most common mutation of the *KRAS* gene is *KRAS* ^*G12D*^, which accounts for 41% of all *KRAS* mutations. The recently developed MRTX1133 is a noncovalent KRAS^G12D^‐selective inhibitor that exerts selective antitumor effects in *KRAS* ^*G12D*^ PDAC cell lines [15, 28]. However, not all PDAC cell lines are sensitive to MRTX1133 treatment [15], and unveiling the mechanism underlying this resistance is urgently needed.

The aim of this study was to elucidate the mechanisms of resistance to KRAS inhibitors and to identify effective combination therapies. Both the KRAS^G12C^ inhibitor sotorasib and the KRAS^G12D^ inhibitor MRTX1133 revealed short‐term resistance mechanisms involving the reactivation of STAT3 and ERK. This resistance was overcome by co‐administration of the MEK inhibitor trametinib and the JAK2 inhibitor fedratinib, leading to significant antitumor effects. Notably, KP‐4, which is resistant to single‐agent therapy with MRTX1133, showed strong antitumor effects when combined with trametinib and fedratinib. Furthermore, we established sotorasib‐resistant and MRTX1133‐resistant cell lines to elucidate the long‐term resistance mechanisms and to find treatment options to overcome this resistance. As expected, the MRTX1133‐resistant cells exhibited activated STAT3 along with ERK and AKT activation. This indicated that the activation of STAT3 and ERK is also involved in the acquisition of long‐term resistance. However, contrary to our expectations, the sotorasib‐resistant cells showed a decrease in STAT3 phosphorylation, while phosphorylation of ERK and AKT increased as expected. Despite the differences in phosphorylated STAT3 expression between sotorasib‐resistant and MRTX1133‐resistant PDAC cells, the resistance was overcome by fedratinib and trametinib in both resistant cells. The difference in STAT3 signaling regulation between sotorasib‐resistant and MRTX1133‐resistant PDAC cells is of great interest, but unfortunately, further validation of this mechanism is currently lacking. Sotorasib is a KRAS‐off inhibitor that binds to the active pocket of inactive KRAS^G12C^ to inhibit its activity [29], whereas MRTX1133 acts on active KRAS^G12D^ and inhibits its activity as an on‐inhibitor [14]. This fundamental difference in the mechanisms of KRAS inhibition may have a relationship to the differences in STAT3 phosphorylation, but further investigation is needed.

A previous study showed that fedratinib reverses KRAS^G12D^‐driven gene signatures [30]. In addition to JAK2, fedratinib blocks other receptor tyrosine kinases, including FLT3, which activates the PI3K/AKT pathway [31]. In our study, the combination of the three drugs not only suppressed ERK and STAT3 phosphorylation but also significantly diminished AKT phosphorylation. The PI3K/AKT pathway has a strong impact on the tumorigenesis of *KRAS*‐mutant PDAC [32], and the co‐inhibition of the RAS/MAPK and PI3K/AKT pathways is effective in treating PDAC [33]. In the present study, MRTX1133‐resistant SUIT‐2 cells did not show strong inhibition of phosphorylated STAT3 expression in the three‐drug combination therapy group compared to that of KP‐4, whereas AKT phosphorylation was significantly impaired, indicating strong antitumor effects. It is reported that knockout of JAK2 increases apoptosis and impairs the proliferation of liver cancer [34], which is consistent with our results of the resected xenograft IHC, showing an increase in TUNEL‐positive cells and a decrease in Ki‐67‐positive cells. These findings indicate that AKT suppression may be a critical factor in the antitumor effect of this three‐drug combination therapy. The three‐drug combination successfully inhibited AKT phosphorylation in all experiments.

Notably, this three‐drug combination therapy significantly reduced the doses of the drugs while maintaining their antitumor effects. The effectiveness of this three‐drug combination therapy was confirmed using the combination index, which is a benchmark for determining whether specific drug combinations show synergistic effects [22]. Combination index values below 0.7 and 0.3 indicate synergism and strong synergism, respectively [23]. Although the combination index for the three‐drug combination therapy was not below 0.3, it was close to this value, suggesting strong synergism. This synergism effect facilitated dose reduction, in contrast to previous reports wherein higher doses of trametinib and fedratinib were required than those used in the present study [35, 36, 37]. Trametinib is reported to be effective in combination with other drugs in *KRAS*‐mutant cancers, and trametinib used in this study is a clinically applicable dose [38]. Dose reduction of fedratinib is especially beneficial because a high dose of fedratinib causes several severe adverse effects, such as grade 3–4 anemia and encephalopathy via unknown mechanisms [39]. The conservation of the body weight of mice and the lack of noticeable adverse effects corroborate the merits of dose reduction.

Furthermore, a novel KRAS G12X inhibitor, RMC‐6236, has recently been developed and is currently undergoing clinical trials [40]. Since mutations in *KRAS* codon 12 are present in >80% of PDAC cases [8], RMC‐6236 benefits the majority of patients with PDAC. Moreover, BI‐3406, a selective SOS1‐KRAS interaction inhibitor, has been developed; dual SOS‐1 and MEK inhibition has shown favorable antitumor effects [41]. BI‐3406 inhibits KRAS expression regardless of the mutation type; therefore, finding an effective combination therapy could hold promise for impacting > 90% of patients with pancreatic cancer. Our study showed that trametinib and fedratinib have synergistic effects with both sotorasib and MRTX1133, suggesting that this combination therapy may be effective regardless of the type of *KRAS* mutation. Therefore, RMC‐6236 and BI‐3406 may be effective when used in combination with trametinib and fedratinib, leading to breakthroughs in unresectable PDAC treatment.

This research, however, is subject to two major limitations. First, we have not elucidated the mechanisms underlying the differences in phosphorylated STAT3 expression between sotorasib‐resistant and MRTX1133‐resistant PDAC cells. Although phosphorylated STAT3 expression was the opposite in sotorasib‐resistant and MRTX‐resistant cells, JAK2 inhibitors were effective in both resistant PDAC cells. This indicates that there are signals regulated by JAK2 other than STAT3 that contribute to the resistance, highlighting the need to investigate these signaling pathways in more detail. If it were possible to obtain multiple PDAC cell lines with *KRAS* ^*G12C*^ mutations, we might have been able to explore the molecular basis of the resistance mechanisms. However, *KRAS* ^*G12C*^ is a relatively rare mutation in PDAC, and there are not any commercially available *KRAS* ^*G12C*^ PDAC cell lines other than MIA PaCa‐2, limiting further investigation. Another limitation is that this combination therapy was not evaluated in immunocompetent models. The combination of immune checkpoint inhibitors and KRAS inhibitors has been reported to be effective in altering the tumor microenvironment [42]. Therefore, the addition of fedratinib makes sense because STAT3 plays an important role in the tumor microenvironment [43]. The possibility of further synergistic effects of fedratinib under immunocompetent conditions should be explored in the future.

## 5 Conclusion

Our results demonstrated that treatment of PDAC cell lines with KRAS inhibitors led to reactivation of the MAPK pathway and increased STAT3 phosphorylation. The combination of MEK and JAK2 inhibitors successfully suppressed this feedback upregulation, showing synergistic inhibitory effects on PDAC cell lines both *in vitro* and *in vivo*. In MRTX1133‐resistant cells, STAT3 phosphorylation increased, whereas in sotorasib‐resistant cell lines, it paradoxically decreased. However, in both cell types, this resistance was overcome by the combined use of MEK and JAK2 inhibitors. The JAK2 inhibitor fedratinib is an FDA‐approved drug with an indication for myelofibrosis, and the MEK inhibitor trametinib is clinically used to treat various cancers, including malignant melanoma and nonsmall cell lung cancer. These agents have established efficacy and safety profiles, making the proposed combination therapy feasible. The three‐drug combination of KRAS inhibitors, MEK inhibitors, and JAK2 inhibitors is a promising strategy against *KRAS*‐mutant PDAC.

## Conflict of interest

The authors declare no conflict of interest.

## Author contributions

Conceptualization: M Kitazawa. Methodology: NH, DK, and YS. Investigation: SM, M Koyama, NH, M Kataoka, and HT. Data curation: SM, YY, M Kataoka, and HT. Data analysis: SM, SN, M Koyama, and YY. Interpretation: SN and M Koyama. Writing—original draft: SM, Writing—critical revision and editing: M Kitazawa, MT, and YS. Supervision: M Kitazawa, M Koyama, NH, MT, DK, and YS. Funding acquisition: M Kitazawa and DK. All authors approved the final manuscript.

### Peer review

The peer review history for this article is available at https://www.webofscience.com/api/gateway/wos/peer‐review/10.1002/1878‐0261.13751.

## Supporting information


**Fig. S1.** Proliferation assay experiment and calculation of combination indices.

**Fig. S2.** Xenograft experiment using SUIT‐2 cells.

**Fig. S3.** Xenograft experiments using MRTX1133‐resistant SUIT‐2 cells.

## Data Availability

The data that support the findings of this study are available from the corresponding author upon reasonable request.

## References

[1] Rahib L , Wehner MR , Matrisian LM , Nead KT . Estimated projection of US cancer incidence and death to 2040. JAMA Netw Open. 2021;4:e214708. 33825840 10.1001/jamanetworkopen.2021.4708 PMC8027914

[2] Tanaka H , Tanaka S , Togawa K , Katanoda K . Practical implications of the update to the 2015 Japan standard population: mortality archive from 1950 to 2020 in Japan. J Epidemiol. 2023;33:372–380. 36775330 10.2188/jea.JE20220302 PMC10257988

[3] Jang JY , Kang MJ , Heo JS , Choi SH , Choi DW , Park SJ , et al. A prospective randomized controlled study comparing outcomes of standard resection and extended resection, including dissection of the nerve plexus and various lymph nodes, in patients with pancreatic head cancer. Ann Surg. 2014;259:656–664. 24368638 10.1097/SLA.0000000000000384

[4] Nichetti F , Rota S , Ambrosini P , Pircher C , Gusmaroli E , Busset MDD , et al. NALIRIFOX, FOLFIRINOX, and gemcitabine with nab‐paclitaxel as first‐line chemotherapy for metastatic pancreatic cancer. JAMA Netw Open. 2024;7:e2350756. 38190183 10.1001/jamanetworkopen.2023.50756 PMC10774994

[5] Conroy T , Desseigne F , Ychou M , Bouché O , Guimbaud R , Bécouarn Y , et al. Groupe Tumeurs digestives of Unicancer, PRODIGE intergroup, FOLFIRINOX versus gemcitabine for metastatic pancreatic cancer. N Engl J Med. 2011;364:1817–1825. 21561347 10.1056/NEJMoa1011923

[6] Von Hoff DD , Ervin T , Arena FP , Chiorean EG , Infante J , Moore M , et al. Increased survival in pancreatic cancer with nab‐paclitaxel plus gemcitabine. N Engl J Med. 2013;369:1691–1703. 24131140 10.1056/NEJMoa1304369 PMC4631139

[7] Moore MJ , Goldstein D , Hamm J , Figer A , Hecht JR , Gallinger S , et al. National Cancer Institute of Canada clinical trials group, erlotinib plus gemcitabine compared with gemcitabine alone in patients with advanced pancreatic cancer: a phase III trial of the National Cancer Institute of Canada clinical trials group. J Clin Oncol. 2007;25:1960–1966. 17452677 10.1200/JCO.2006.07.9525

[8] Waters AM , Der CJ . KRAS: the critical driver and therapeutic target for pancreatic cancer. Cold Spring Harb Perspect Med. 2018;8:a031435. 29229669 10.1101/cshperspect.a031435 PMC5995645

[9] Golan T , Hammel P , Reni M , Van Cutsem E , Macarulla T , Hall MJ , et al. Maintenance Olaparib for germline BRCA‐mutated metastatic pancreatic cancer. N Engl J Med. 2019;381:317–327. 31157963 10.1056/NEJMoa1903387 PMC6810605

[10] Friedenson B . BRCA1 and BRCA2 pathways and the risk of cancers other than breast or ovarian. MedGenMed. 2005;7:60. PMC1681605 16369438

[11] Hong DS , DuBois SG , Kummar S , Farago AF , Albert CM , Rohrberg KS , et al. Larotrectinib in patients with TRK fusion‐positive solid tumours: a pooled analysis of three phase 1/2 clinical trials. Lancet Oncol. 2020;21:531–540. 32105622 10.1016/S1470-2045(19)30856-3 PMC7497841

[12] Allen MJ , Zhang A , Bavi P , Kim JC , Jang GH , Kelly D , et al. Molecular characterisation of pancreatic ductal adenocarcinoma with NTRK fusions and review of the literature. J Clin Pathol. 2023;76:158–165. 34583947 10.1136/jclinpath-2021-207781

[13] Canon J , Rex K , Saiki AY , Mohr C , Cooke K , Bagal D , et al. The clinical KRAS(G12C) inhibitor AMG 510 drives anti‐tumour immunity. Nature. 2019;575:217–223. 31666701 10.1038/s41586-019-1694-1

[14] Wang X , Allen S , Blake JF , Bowcut V , Briere DM , Calinisan A , et al. Identification of MRTX1133, a noncovalent, potent, and selective KRASG12DInhibitor. J Med Chem. 2022;65:3123–3133. 34889605 10.1021/acs.jmedchem.1c01688

[15] Hallin J , Bowcut V , Calinisan A , Briere DM , Hargis L , Engstrom LD , et al. Anti‐tumor efficacy of a potent and selective non‐covalent KRASG12D inhibitor. Nat Med. 2022;28:2171–2182. 36216931 10.1038/s41591-022-02007-7

[16] Ou S‐HI , Jänne PA , Leal TA , Rybkin II , Sabari JK , Barve MA , et al. First‐in‐human phase I/IB dose‐finding study of Adagrasib (MRTX849) in patients with advanced KRASG12C solid tumors (KRYSTAL‐1). J Clin Oncol. 2022;40:2530–2538. 35167329 10.1200/JCO.21.02752 PMC9362872

[17] Awad MM , Liu S , Rybkin II , Arbour KC , Dilly J , Zhu VW , et al. Acquired resistance to KRASG12C inhibition in cancer. N Engl J Med. 2021;384:2382–2393. 34161704 10.1056/NEJMoa2105281 PMC8864540

[18] Ryan MB , Fece de la Cruz F , Phat S , Myers DT , Wong E , Shahzade HA , et al. Vertical pathway inhibition overcomes adaptive feedback resistance to KRASG12C inhibition. Clin Cancer Res. 2020;26:1633–1643. 31776128 10.1158/1078-0432.CCR-19-3523 PMC7124991

[19] Yaeger R , Weiss J , Pelster MS , Spira AI , Barve M , Ou S‐HI , et al. Adagrasib with or without cetuximab in colorectal cancer with mutated KRAS G12C. N Engl J Med. 2023;388:44–54. 36546659 10.1056/NEJMoa2212419 PMC9908297

[20] Hondo N , Kitazawa M , Koyama M , Nakamura S , Tokumaru S , Miyazaki S , et al. MEK inhibitor and anti‐EGFR antibody overcome sotorasib resistance signals and enhance its antitumor effect in colorectal cancer cells. Cancer Lett. 2023;567:216264. 37336286 10.1016/j.canlet.2023.216264

[21] Nagathihalli NS , Castellanos JA , Lamichhane P , Messaggio F , Shi C , Dai X , et al. Inverse correlation of STAT3 and MEK signaling mediates resistance to Ras pathway inhibition in pancreatic cancer. Cancer Res. 2018;78:6235–6246. 30154150 10.1158/0008-5472.CAN-18-0634 PMC6878978

[22] Chou TC , Talalay P . Quantitative analysis of dose‐effect relationships: the combined effects of multiple drugs or enzyme inhibitors. Adv Enzym Regul. 1984;22:27–55. 10.1016/0065-2571(84)90007-4 6382953

[23] Chou T‐C . Theoretical basis, experimental design, and computerized simulation of synergism and antagonism in drug combination studies. Pharmacol Rev. 2006;58:621–681. 16968952 10.1124/pr.58.3.10

[24] Siegel RL , Miller KD , Wagle NS , Jemal A . Cancer statistics. CA Cancer J Clin. 2023;73(2023):17–48. 36633525 10.3322/caac.21763

[25] National Cancer Institute . Pancreatic cancer — cancer stat facts. n.d. https://seer.cancer.gov/statfacts/html/pancreas.html (accessed January 9, 2024).

[26] Schnelldorfer T , Ware AL , Sarr MG , Smyrk TC , Zhang L , Qin R , et al. Long‐term survival after pancreatoduodenectomy for pancreatic adenocarcinoma: is cure possible? Ann Surg. 2008;247:456–462. 18376190 10.1097/SLA.0b013e3181613142

[27] Hruban RH , Goggins M , Parsons J , Kern SE . Progression model for pancreatic cancer. Clin Cancer Res. 2000;6:2969–2972. 10955772

[28] Gulay KCM , Zhang X , Pantazopoulou V , Patel J , Esparza E , Pran Babu DS , et al. Dual inhibition of KRASG12D and pan‐ERBB is synergistic in pancreatic ductal adenocarcinoma. Cancer Res. 2023;83:3001–3012. 37378556 10.1158/0008-5472.CAN-23-1313 PMC10502451

[29] Lanman BA , Allen JR , Allen JG , Amegadzie AK , Ashton KS , Booker SK , et al. Discovery of a covalent inhibitor of KRASG12C (AMG 510) for the treatment of solid tumors. J Med Chem. 2020;63:52–65. 31820981 10.1021/acs.jmedchem.9b01180

[30] Liu LW , Hsieh YY , Yang PM . Bioinformatics data mining repurposes the JAK2 (Janus kinase 2) inhibitor fedratinib for treating pancreatic ductal adenocarcinoma by reversing the KRAS (kirsten rat sarcoma 2 viral oncogene homolog)‐driven gene signature. J Pers Med. 2020;10:1–17. 10.3390/jpm10030130 PMC7563462 32947833

[31] Mizuki M , Fenski R , Halfter H , Matsumura I , Schmidt R , Müller C , et al. Flt3 mutations from patients with acute myeloid leukemia induce transformation of 32D cells mediated by the Ras and STAT5 pathways. Blood. 2000;96:3907–3914. 11090077

[32] Schmidt KM , Hellerbrand C , Ruemmele P , Michalski CW , Kong B , Kroemer A , et al. Inhibition of mTORC2 component RICTOR impairs tumor growth in pancreatic cancer models. Oncotarget. 2017;8:24491–24505. 28445935 10.18632/oncotarget.15524 PMC5421865

[33] Brown WS , McDonald PC , Nemirovsky O , Awrey S , Chafe SC , Schaeffer DF , et al. Overcoming adaptive resistance to KRAS and MEK inhibitors by Co‐targeting mTORC1/2 complexes in pancreatic cancer. Cell Rep Med. 2020;1:100131. 33294856 10.1016/j.xcrm.2020.100131 PMC7691443

[34] Xu Y , Lv S‐X . The effect of JAK2 knockout on inhibition of liver tumor growth by inducing apoptosis, autophagy and anti‐proliferation via STATs and PI3K/AKT signaling pathways. Biomed Pharmacother. 2016;84:1202–1212. 27788478 10.1016/j.biopha.2016.09.040

[35] Kawaguchi K , Igarashi K , Murakami T , Kiyuna T , Lwin TM , Hwang HK , et al. MEK inhibitors cobimetinib and trametinib, regressed a gemcitabine‐resistant pancreatic‐cancer patient‐derived orthotopic xenograft (PDOX). Oncotarget. 2017;8:47490–47496. 28537897 10.18632/oncotarget.17667 PMC5564580

[36] Wernig G , Kharas MG , Okabe R , Moore SA , Leeman DS , Cullen DE , et al. Efficacy of TG101348, a selective JAK2 inhibitor, in treatment of a murine model of JAK2V617F‐induced polycythemia Vera. Cancer Cell. 2008;13:311–320. 18394554 10.1016/j.ccr.2008.02.009

[37] Geron I , Abrahamsson AE , Barroga CF , Kavalerchik E , Gotlib J , Hood JD , et al. Selective inhibition of JAK2‐driven erythroid differentiation of polycythemia Vera progenitors. Cancer Cell. 2008;13:321–330. 18394555 10.1016/j.ccr.2008.02.017

[38] Corcoran RB , Do KT , Kim JE , Cleary JM , Parikh AR , Yeku OO , et al. Phase I/II study of combined BCL‐xL and MEK inhibition with navitoclax and trametinib in KRAS or NRAS mutant advanced solid tumors. Clin Cancer Res. 2024;30:1739–1749. 38456660 10.1158/1078-0432.CCR-23-3135 PMC11061595

[39] Pardanani A , Harrison C , Cortes JE , Cervantes F , Mesa RA , Milligan D , et al. Safety and efficacy of fedratinib in patients with primary or secondary myelofibrosis: a randomized clinical trial. JAMA Oncol. 2015;1:643–651. 26181658 10.1001/jamaoncol.2015.1590

[40] Arbour KC , Punekar S , Garrido‐Laguna I , Hong DS , Wolpin B , Pelster MS , et al. 652O preliminary clinical activity of RMC‐6236, a first‐in‐class, RAS‐selective, tri‐complex RAS‐MULTI(ON) inhibitor in patients with KRAS mutant pancreatic ductal adenocarcinoma (PDAC) and non‐small cell lung cancer (NSCLC). Ann Oncol. 2023;34:S458.

[41] Hofmann MH , Gmachl M , Ramharter J , Savarese F , Gerlach D , Marszalek JR , et al. BI‐3406, a potent and selective SOS1‐KRAS interaction inhibitor, is effective in KRAS‐driven cancers through combined MEK inhibition. Cancer Discov. 2021;11:142–157. 32816843 10.1158/2159-8290.CD-20-0142 PMC7892644

[42] Briere DM , Li S , Calinisan A , Sudhakar N , Aranda R , Hargis L , et al. The KRASG12C inhibitor MRTX849 reconditions the tumor immune microenvironment and sensitizes tumors to checkpoint inhibitor therapy, Mol Cancer Ther. 2021;8:975–985. 10.1158/1535-7163.MCT-20-0462 PMC8444277 33722854

[43] Bournazou E , Bromberg J . Targeting the tumor microenvironment: JAK‐STAT3 signaling. JAKSTAT. 2013;2:e23828. 24058812 10.4161/jkst.23828 PMC3710325

