# Supplementary figures and images for: Targeting KRAS‐mutant pancreatic cancer through simultaneous inhibition of KRAS, MEK, and JAK2
Source: Mol Oncol. 2024 Oct 14;19(2):377–90. doi: 10.1002/1878-0261.13751 (PMC11793007; doi:10.1002/1878-0261.13751)

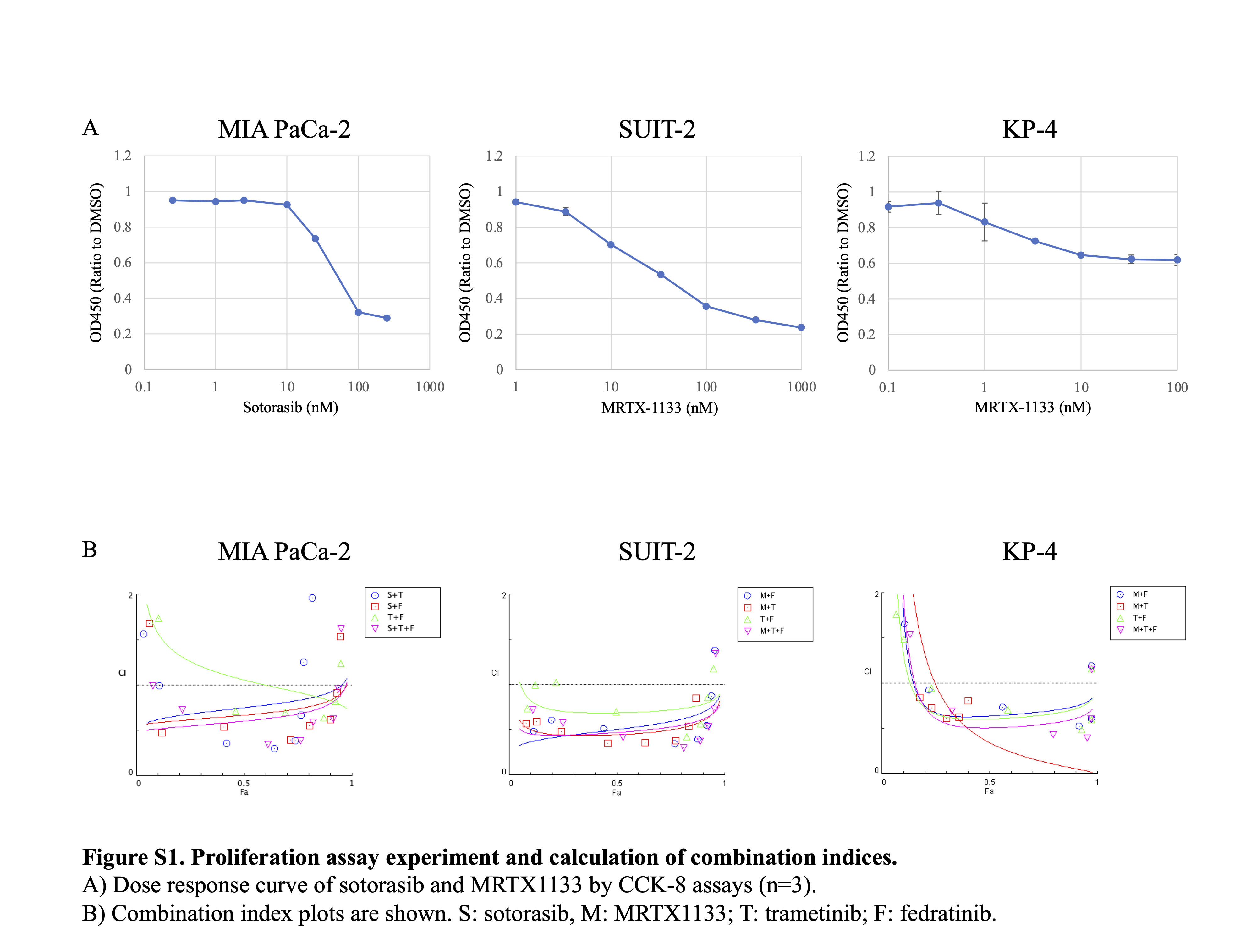

Supplement: Supplementary file 1 — Fig. S1. Proliferation assay experiment and calculation of combination indices. [file MOL2-19-377-s002.tiff]

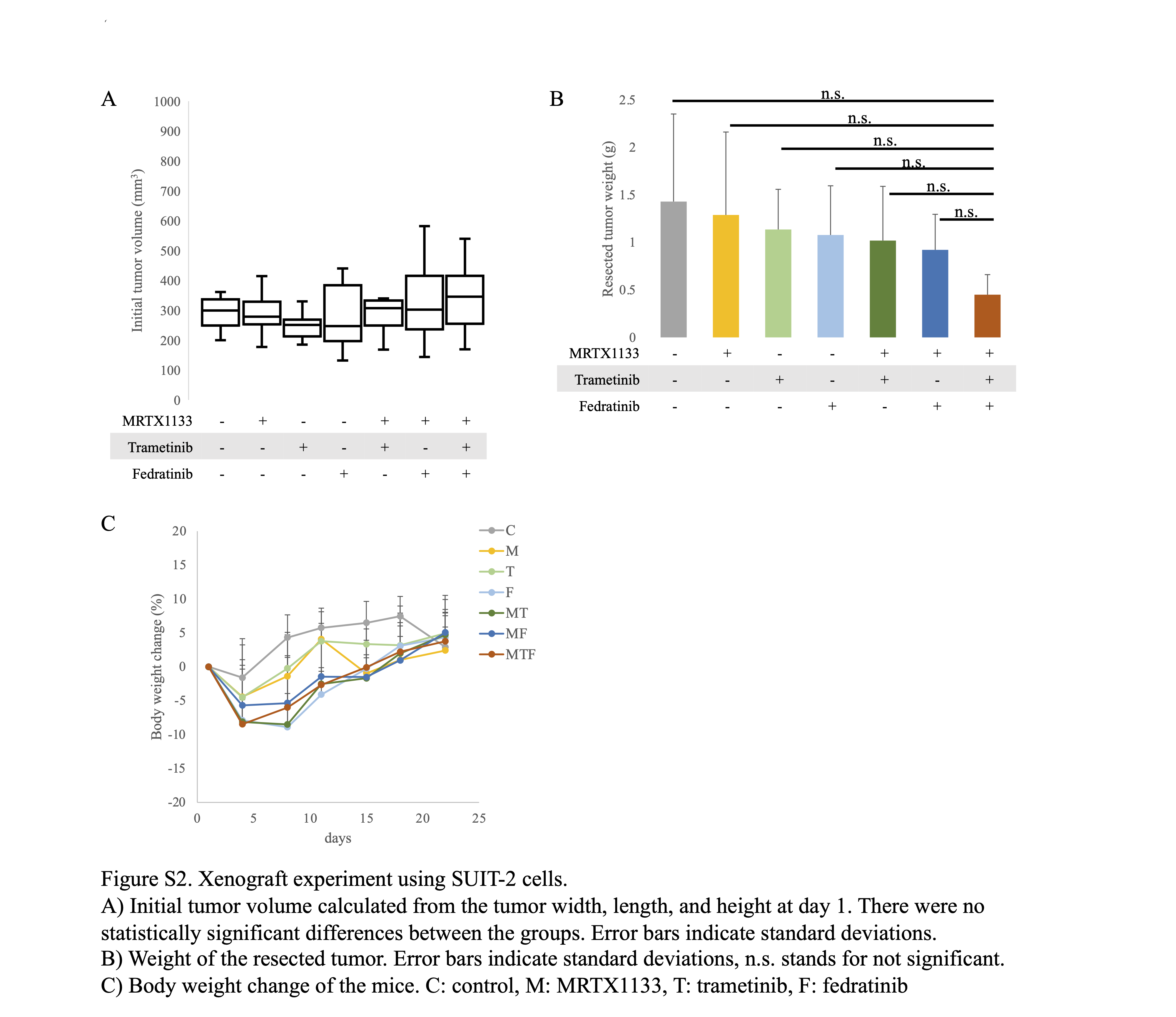

Supplement: Supplementary file 2 — Fig. S2. Xenograft experiment using SUIT‐2 cells. [file MOL2-19-377-s003.tiff]

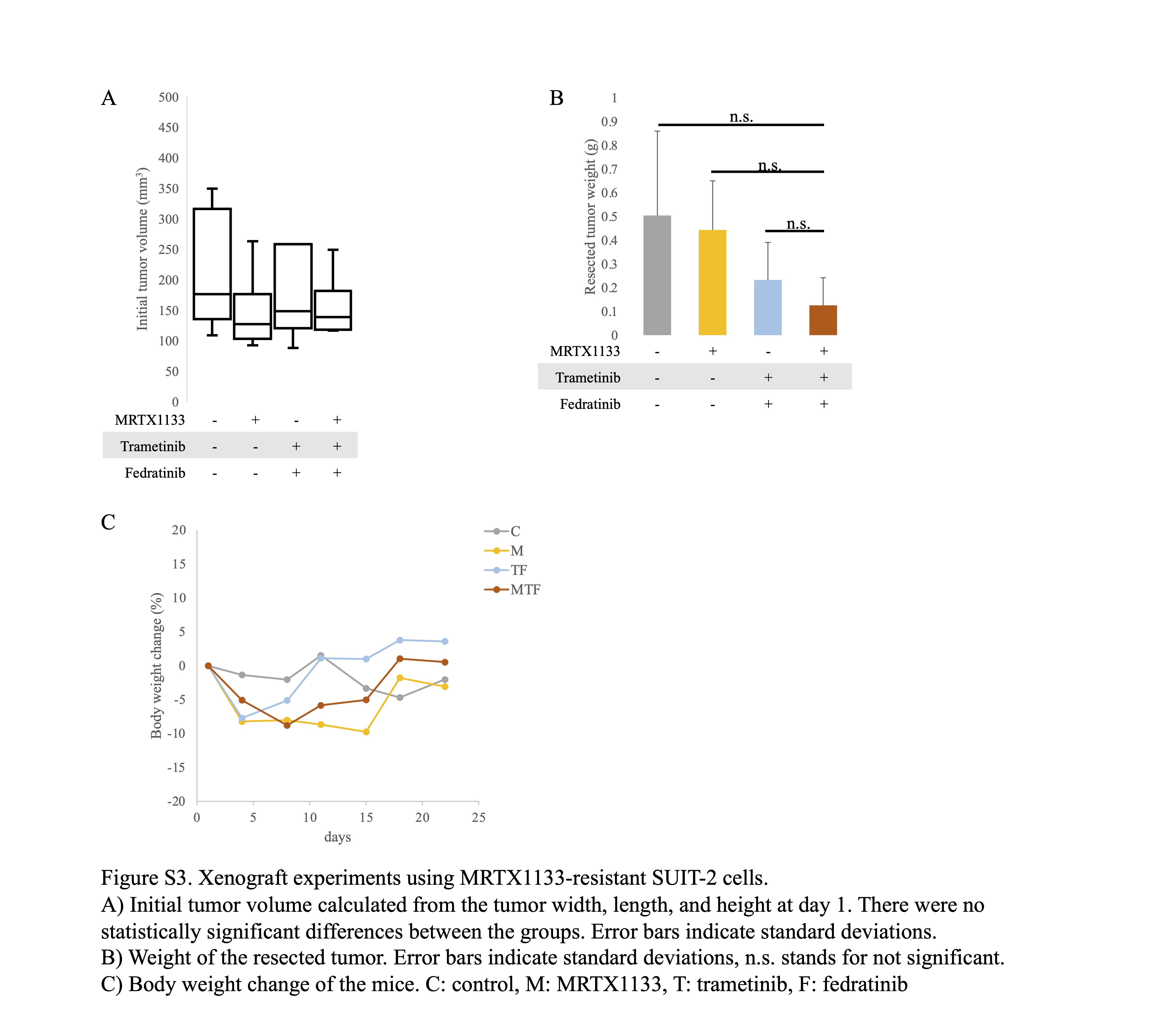

Supplement: Supplementary file 3 — Fig. S3. Xenograft experiments using MRTX1133‐resistant SUIT‐2 cells. [file MOL2-19-377-s001.tiff]
